# The chromosome-scale genome and population genomics reveal the adaptative evolution of *Populus pruinosa* to desertification environment

**DOI:** 10.1093/hr/uhae034

**Published:** 2024-02-06

**Authors:** Jianhao Sun, Jindong Xu, Chen Qiu, Juntuan Zhai, Shanhe Zhang, Xiao Zhang, Zhihua Wu, Zhijun Li

**Affiliations:** College of Life Science and Technology, Tarim University, Aral 843300, China; Xinjiang Production & Construction Corps Key Laboratory of Protection and Utilization of Biological Resources in Tarim Basin, Aral 843300, China; Desert Poplar Research Center of Tarim University, Aral 843300, China; College of Life Sciences, South-Central Minzu University, Wuhan 430074, China; College of Life Science and Technology, Tarim University, Aral 843300, China; Xinjiang Production & Construction Corps Key Laboratory of Protection and Utilization of Biological Resources in Tarim Basin, Aral 843300, China; Desert Poplar Research Center of Tarim University, Aral 843300, China; College of Life Science and Technology, Tarim University, Aral 843300, China; Xinjiang Production & Construction Corps Key Laboratory of Protection and Utilization of Biological Resources in Tarim Basin, Aral 843300, China; Desert Poplar Research Center of Tarim University, Aral 843300, China; College of Life Science and Technology, Tarim University, Aral 843300, China; Xinjiang Production & Construction Corps Key Laboratory of Protection and Utilization of Biological Resources in Tarim Basin, Aral 843300, China; Desert Poplar Research Center of Tarim University, Aral 843300, China; College of Life Science and Technology, Tarim University, Aral 843300, China; Xinjiang Production & Construction Corps Key Laboratory of Protection and Utilization of Biological Resources in Tarim Basin, Aral 843300, China; Desert Poplar Research Center of Tarim University, Aral 843300, China; College of Life Sciences, Zhejiang Normal University, Jinhua 321004, China; College of Life Science and Technology, Tarim University, Aral 843300, China; Xinjiang Production & Construction Corps Key Laboratory of Protection and Utilization of Biological Resources in Tarim Basin, Aral 843300, China; Desert Poplar Research Center of Tarim University, Aral 843300, China

## Abstract

The *Populus pruinosa* is a relic plant that has managed to survive in extremely harsh desert environments. Owing to intensifying global warming and desertification, research into ecological adaptation and speciation of *P. pruinosa* has attracted considerable interest, but the lack of a chromosome-scale genome has limited adaptive evolution research. Here, a 521.09 Mb chromosome-level reference genome of *P. pruinosa* was reported. Genome evolution and comparative genomic analysis revealed that tandemly duplicated genes and expanded gene families in *P. pruinosa* contributed to adaptability to extreme desert environments (especially high salinity and drought). The long terminal repeat retrotransposons (LTR-RTs) inserted genes in the gene body region might drive the adaptive evolution of *P. pruinosa* and species differentiation in saline-alkali desert environments. We recovered genetic differentiation in the populations of the northern Tianshan Mountain and southern Tianshan Mountain through whole-genome resequencing of 156 *P. pruinosa* individuals from 25 populations in China. Further analyses revealed that precipitation drove the local adaptation of *P. pruinosa* populations via some genetic sites, such as MAG2-interacting protein 2 (*MIP2*) and SET domain protein 25 (*SDG25*). This study will provide broad implications for adaptative evolution and population studies by integrating internal genetic and external environmental factors in *P. pruinosa*.

## Introduction

**Figure 1:**
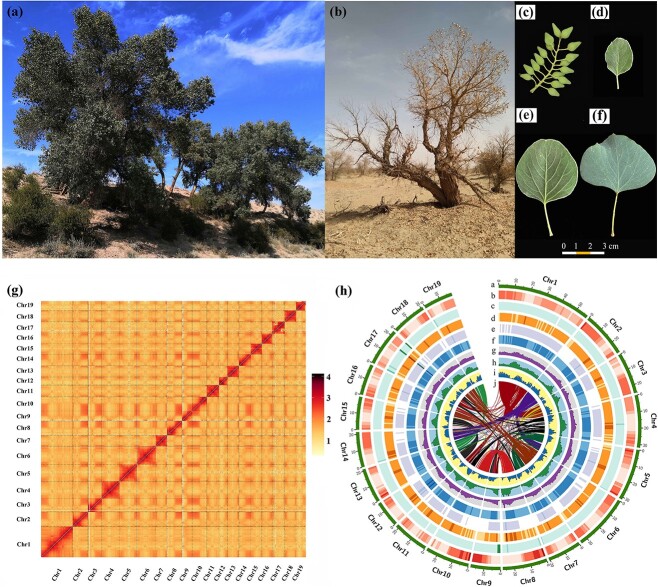
Morphological characteristics and genome overview (v2.0) of the *Populus pruinosa*. (**a**)**,** (**b**) *P. pruinosa* appearance in summer and winter. (**c**) Shoot and fruit. (**d**) Long-oval leaves. (**e**) Ovate leaves. (**f**) Broad-ovate leaves. (**g**) The Hi-C heatmap at 100-kb resolution of *P. pruinosa* genome assembly. Chr1-Chr19 represented the 19 chromosomes. (**h**) Circos plot of *P. pruinosa* genome assembly. (a) Assembled 19 chromosomes. (b–i) The distribution of the gene density, GC density, transposon density, tandem repeat density, SSR density, LTR density, Gypsy density, and Copia density, respectively, with densities calculated in 1-Mb windows. (j) Relationship between syntenic blocks, as indicated by lines.

Poplars have economic and ecological importance because of their amenability to *in vitro* regeneration and fast vegetative reproduction throughout the Northern Hemisphere. Hence, they are usually used as model forest trees for various studies [1–3]. *Populus pruinosa* Schrenk (2n = 38), distributed in highly saline-alkali environments in western China’s central deserts and certain central Asian countries, exhibits a narrow ecological niche compared to its sister species (*Populus euphratica* Oliv), reaching heights of up to 20 m with long oval, ovate, or broad-ovate leaves adorned with thick hairs [4, 5] (Fig. 1a–f). While *P. pruinosa* and *P. euphratica* both inhabit Central and Western Asia, *P. euphratica* predominates in less saline deserts, whereas *P. pruinosa* thrives in highly saline deserts [6, 7]. Therefore, *P. pruinosa* is an excellent desert species for research into species differentiation and genetic adaptation. However, the genetic basis of *P. pruinosa*’s adaptation to desert climates is limited because of insufficient genomic resources.

The habitat of *P. pruinosa* forests is facing significant degradation in China, and genetic diversity has been reduced by increased habitat destruction and human activity [8]. Considering the high mountainous barrier created by the Tianshan Mountains in the middle of Xinjiang, running roughly east–west for approximately 2500 km, *P. pruinosa* forests show discontinuous geographical distribution or environmental heterogeneity. Environmental heterogeneity, especially climate change, places different levels of selective pressure on plants, which in turn drives localized adaptation in the natural population [9, 10]. However, the impact of environmental heterogeneity on population differentiation and local adaptation of *P. pruinosa* in China remains poorly understood.

Despite the release of the *P. pruinosa* draft genome [11], its high fragmentation caused by short-read assembly has hindered its use in comparative genomics, phylogenomics, and population genomics [12]. The chromosome-level genome of *Populus deltoides* drives the study of its sex-determining mechanisms and key regulatory genes for female fluffy catkins [13]. The chromosome-level genome of *Populus tremula* advances the study of the aspen evolutionary history and the genetic mechanisms of local adaptation [14]. Here, a chromosome-level *P. pruinosa* genome was assembled and annotated by using integrated approaches, namely, Illumina, Pacbio, and high-throughput chromatin conformation capture (Hi-C). Then, we performed the whole-genome resequencing (WGS) for 156 *P. pruinosa* accessions from China to reveal its adaptative evolution and genetic differentiation. Our aim was to: (i) explore the evolutionary pattern of *P. pruinosa* by using a chromosome-scale reference genome; and (ii) investigate the population structure and genomic underpinnings of climate adaptation of *P. pruinosa* in China. This study would act as a crucial guide for future research on the genomics-assisted adaptive evolution and genetic improvement of *P. pruinosa*.

## Results

### *De novo* assembly of *P. pruinosa* genome

The genome of a female *P. pruinosa* was sequenced and assembled, with an estimated size of 583.98 Mb (Fig. S1, see online supplementary material). A k-mer with a length of 17 indicated that the genome had low heterozygosity (0.96%) and a repetitive sequence content of 59.54% (Table S1, see online supplementary material). A comprehensive *de novo* assembly strategy combining 30.7 Gb (52.6 X) Illumina paired-end reads, 39.5 Gb (66.8 X) PacBio single-molecule long reads and 72.8 Gb (124.7 X) Hi-C reads pairs was adopted (Fig. S2, Table S2, see online supplementary material). The resulting assembly using integrated technologies of Illumina and Pacbio consisted of 676 contigs, with a contig N50 of 20.96 Mb (Table S3, see online supplementary material). Utilizing Hi-C interaction data, the contig N50 was further improved to 21.06 Mb, 521.09 Mb contig sequences were obtained, and 480.44 Mb contig sequences were anchored onto 19 chromosomes (Table 1 and Fig. 1g and h; Table S4, see online supplementary material). The genome in this study was defined as a version of v2.0 compared to the *P. pruinosa* draft genome v1.0. The new assembled genome represented a 1503-fold improvement in contiguity compared to the genome v1.0 (contigs N50: 21.06 Mb versus 14.01 kb).

**Table 1:** Comparison of the *de novo* assembled *Populus pruinosa* genome (v2.0) with the *P. pruinosa* draft genome (v1.0).

**Category**	***P. pruinosa* genome (v2.0)**	***P. pruinosa* genome (v1.0)**
Assembly size (bp)	521 092 247	479 307 600
GC content	33.00%	31.80%
Repeat content	50.56%	45.47%
**Genome assembly**		
Number of contigs	676	170 219
Number of scaffolds	636	78 960
Contigs N50 (bp)	21 063 123	14 011
BUSCO	97.70%	98.00%
LTR Assembly Index (LAI)	15.13	3.84
Merqury (QV)	43.19	
**Genome annotation**		
Number of protein-coding genes	33 291	35 131
Average transcript length (bp)	3106.76	3703.40
Average exon length (bp)	230.71	226.27
Average intron length (bp)	464.21	561.98
**Number of non-coding RNA**		
miRNAs	523	
tRNAs	2363	
rRNAs	7632	
snRNAs	951	
**Number of functionally annotated genes**		
Total	32 011	30 938

The clean Illumina short reads were aligned to the assembled *P. pruinosa* genome using BWA, resulting in a mapping rate and coverage of 98.49% and 99.84%, respectively (Table S5, see online supplementary material). The analysis of Benchmarking Universal Single-Copy Orthologs (BUSCO) revealed that 97.7% (1577) of the gene models exhibited completeness in our newly assembled genome (Table 1;Table S6, see online supplementary material). The LAI (LTR Assembly Index) score of an assembly constructed by Hi-C was 15.1, reaching the criterion of reference quality [15]. Furthermore, Merqury results showed that the quality value (QV) = 43.19, and the error rate was only 0.005%. The obtained results provide strong evidence for the high quality and integrity of the new *P. pruinosa* genome.

### Genome annotation of *P. pruinosa*

A total of 33 291 protein-coding genes were predicted (Table 1). Among them, 32 011 genes were annotated by at least one public database (Table S7, see online supplementary material). The gene density on chromosome 9 and chromosome 17 was the highest (86.40 genes/Mb) and lowest (47.98 genes/Mb), respectively (Table S8, see online supplementary material). Moreover, 523 miRNAs, 2363 tRNAs, 7632 rRNAs, and 951 snRNAs were identified (Table 1). Approximately 50.56% of the *P. pruinosa* genome (v2.0) was identified as 263.47 Mb repeat sequences, and transposable elements (TEs) occupied 49.26% (256.7 Mb) of the genome assembly length (Fig. 1h; Table S9, see online supplementary material).

### Genome evolution of *P. pruinosa*

**Figure 2:**
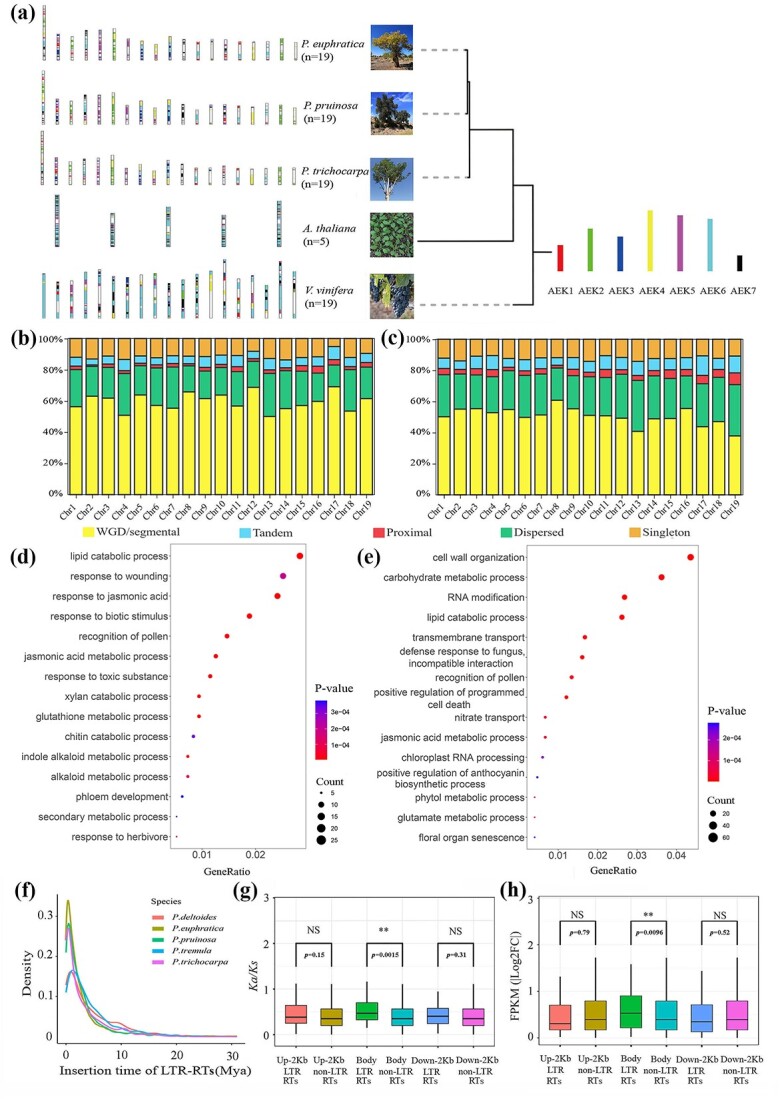
Genome evolution of *Populus pruinosa*. (**a**) Evolutionary scenario of the five dicotyledons (*P. pruinosa*, *Populus euphratica*, *Populus trichocarpa*, *Arabidopsis thaliana*, and *Vitis vinifera*) from the ancestral eudicot karyotype (AEK) of seven protochromosomes. (**b, c**) Classification of gene duplicates origin in the genomes of *P. pruinosa* and *P. euphratica*. The origins of gene duplicates were classified into five types: WGD/segmental duplication, tandem duplication, proximal duplication, dispersed duplication, and singleton. (**d, e**) Biological preference of tandem duplicated genes in *P. pruinosa* and *P. euphratica* (biological process category). (**f**) Insertion time of LTR-RTs in five *Populus* species. (**g**) Ka/Ks distribution of orthologous genes between *P. pruinosa* and *P. euphratica* in LTR-RTs-inserted genes patterns (upstream 2 kb, gene body, and downstream 2 kb). (**h**) Gene expression changes of orthologous genes between *P. pruinosa* and *P. euphratica*. Up-2 kb LTR-RTs, Body LTR-RTs, and Down-2 kb LTR-RTs represented the LTR-RTs-inserted genes in upstream 2 kb of genes, LTR-RTs-inserted genes in the gene body, and LTR-RTs-inserted genes in downstream 2 kb of genes, respectively. Up-2 kb non-LTR-RTs, Body non-LTR-RTs, and Down-2 kb non-LTR-RTs represented the non-LTR-RTs-inserted genes in upstream 2 kb of genes, non-LTR-RTs-inserted genes in the gene body, and non-LTR-RTs-inserted genes in downstream 2 kb of genes, respectively. The asterisks denote significant differences identified through the Wilcox test (^**^P < 0.01).

For the evolutionary study of the *P. pruinosa* genome, a comparative analysis was conducted among five species with chromosome-level genomes and an ancestral eudicot karyotype (AEK) genome [16]. On the basis of the AEK genome, 6828 genes (25.9%) were identified in *Vitis vinifera*, 14 893 genes (52.1%) in *Arabidopsis thaliana*, 12 857 genes (21.93%) in *Populus trichocarpa*, 12 847 genes (29.8%) in *P. euphratica*, and 12 925 genes (33.59%) in *P. pruinosa* (Fig. 2a). These findings implied that the chromosomes of these lineages experienced varying degrees of multiple rearrangements post their divergence from the AEK genome. Notably, the chromosome 18 of poplars (*P. trichocarpa*, *P. euphratica*, and *P. pruinosa*) showed collinearity only with AEK chromosome 2, indicating that poplar chromosome 18 is relatively ancient and mainly originated from AEK chromosome 2.

To gain further insights into *P. pruinosa*’s evolutionary events, we analysed and compared the types of duplicated genes in its genome with that of *P. euphratica* (Table S10, see online supplementary material). The results suggested that whole genome duplication (WGD)/segmental duplication in *P. pruinosa* (58.0%) and *P. euphratica* (51.42%) accounted for the majority of gene duplication compared with the other three types: dispersed duplication, tandem duplication, and proximal duplication (Fig. 2b and c). Subsequently, functional enrichment analysis was employed to assess whether genes of different duplication origins exhibit preferential biological functionality (Figs. S3 and S4, see online supplementary material). As sister species, the biological functions (e.g. ‘response to water deprivation’, ‘flower development’, and ‘response to light stimulus’) of genes originating from WGD in *P. pruinosa* and *P. euphratica* showed clear congruence (Figs S3a and Figs S4a, see online supplementary material). This result suggested that *P. pruinosa* and *P. euphratica* maintained genetic stability during the evolutionary process, and the preserved genes laid an adaptive foundation for the survival and reproduction of desert poplars. Tandem duplication is the primary force driving the expansion of defence-responsive genes for extensive adaptability in plants to complex environments [17, 18]. Interestingly, except for ‘jasmonic acid metabolic process’ and ‘lipid catabolic process’, the tandemly replicated genes in *P. pruinosa* and *P. euphratica* showed significantly different biological preferences (Fig. 2d and e). The biological preferences of genes originating from tandem duplication in *P. euphratica* were related to ‘RNA modification’, ‘defense response to fungus’, ‘cell wall organization’, and ‘transmembrane transport’. The biological preferences of genes originating from tandem duplication in *P. pruinosa* were mainly related to biotic and abiotic stress responses (e.g. ‘response to biotic stimulus’, ‘response to toxic substance’, ‘glutathione metabolic process’, and ‘response to herbivore’). Of these, *HS1* (heat stabilising protein 1, PprTF10G0924.1, PprTF10G0925.1, PeuTF10G01042.1, and PeuTF10G01043.1) was associated with antimicrobial activity [19] and was tandemly duplicated in both *P. euphratica* and *P. pruinosa* (Table S11, see online supplementary material). In addition, tandemly replicated *RCI2A* (low-temperature and salt-responsive protein family, PprTF05G0019.1 and PprTF05G0020.1), *ERD4* (early response protein to dehydration stress, PprTF01G3034. 1, and PprTF01G3035.1), and *GSTF6* (glutathione S-transferase 6, PprTF02G1860.1, and PprTF02G1859.1) were only present in *P. pruinosa* and co-upregulated under salt stress and/or drought stress. It was found that they both were involved in regulating plant tolerance to salt stress and dehydration stress [20–24]. These findings suggested that desert poplars might undergo distinct adaptive evolutionary processes post-speciation, with tandem duplication events playing a significant role in the evolution of *P. pruinosa.*

**Figure 3:**
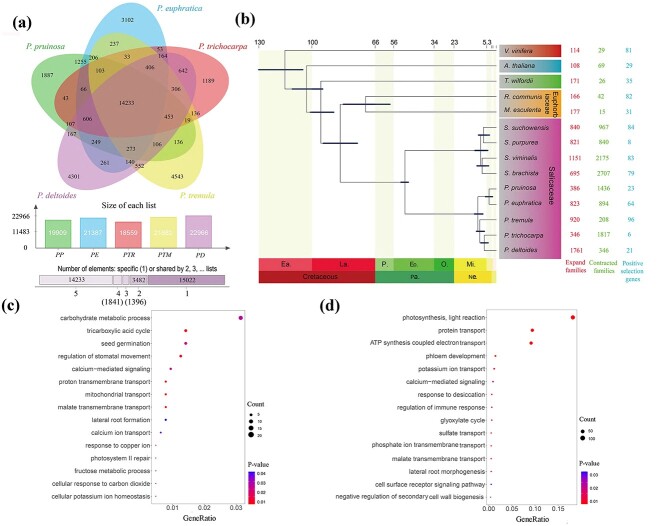
Comparative genomic analysis of *Populus pruinosa*. (**a**) Venn diagram of gene families across five *Populus* species. (**b**) Phylogenetic trees and gene family evolution in 14 species. Pink indicated expansions, green indicated contractions, and blue indicated positive selections. (**c**) Biological preference of unique gene families in *P. pruinosa*. (**d**) Biological preference of expanded gene families in *P. pruinosa*.

To gain a deeper understanding of the amplification history of repetitive elements and their impact on gene expression, 3439 intact LTR retrotransposons (LTR-RTs) were annotated in the *P. pruinosa* genome (Table S12, see online supplementary material). Analysis of insertion time indicated that a burst of LTR-RT activity occurred during the last 10 million years in five *Populus* species, and the proliferation of LTR-RTs in *P. pruinosa* peaked ∼0.45 million years ago (Mya) (Fig. 2f). Moreover, to investigate the insertion preference of transposons in adjacent regions around genes, gene-adjacent region was partitioned into three segments: a gene body and upstream and downstream 2 kb regions (non-transcribed regions). The number of LTR-RTs inserted into the gene body was less than that in non-transcribed regions (Fig. S5, see online supplementary material). Further investigation indicated that the LTR-RT inserted genes in the gene body region showed markedly increased protein evolutionary rates (Ka/Ks) and a greater degree of differential expression than the non-LTR-RT inserted genes between the orthologous genes in *P. pruinosa* and *P. euphratica* (Fig. 2g and h; Table S13, see online supplementary material). These results indicated the rapid divergence and adaptive evolution of the LTR-RT-inserted genes, including *VRN1* (AP2/B3-like transcriptional factor family, PprTF14G1176.1), which promotes rapid flowering [25]; *DEG5* (DEGP protease 5, PprTF04G0499.1), which is involved in the repair of photosystem II and protection against photoinhibition [26]; and *NAC96* (NAC domain containing protein 96, PprTF13G0664.1), which regulates resistance to dehydration and osmotic stress as a positive regulator of ABA-responsive signalling [27]. Overall, the rapid amplification of repetitive elements in *P. pruinosa* held significant implications for the genomic adaptive evolution and the species differentiation.

### Comparative genomics analysis of *P. pruinosa* genome

To explain the evolutionary history and genetic relationship of *P. pruinosa*, its genome was compared with other Dicotyledoneae species. A total of 28 804 orthogroups were identified. *P. pruinosa* shared 9402 gene families (18 231 genes) with the 13 species (Fig. S6, see online supplementary material). For subsequent phylogenetic analysis, 626 single-copy gene families were employed. The result showed that the divergence time between *P. pruinosa* and *P. euphratica* was approximately 3.94 (2.10–5.82) Mya (Fig. 3b), which coincides with the time period of the mountain uplift in Asia during the Pliocene and was accompanied by a trend towards the aridification of the climate [28, 29].

To acquire insights into the genomic foundation of environmental adaptation, genomic sequences analysis of *P. pruinosa* and its close relatives were performed (Fig. 3a). We found 1255 gene families common to *P. pruinosa* and *P. euphratica* alone, which were crucial in balancing stress tolerance (e.g. ‘cellular response to water deprivation’ and ‘cell redox homeostasis’) and growth and development (e.g. ‘regulation of root meristem growth’ and ‘wax biosynthetic process’) (Fig. S7a and Table S14, see online supplementary material). Gene families present only in *P. euphratica* might contribute to its survival in more arid (e.g. ‘response to water deprivation’ and ‘regulation of stomatal closure’) and higher latitude (‘cellular response to cold’) regions (Fig. S7b and Table S15, see online supplementary material). The gene families present only in *P. pruinosa* are mainly related to ion homeostasis (e.g. ‘calcium ion transport’ and ‘cellular potassium ion homeostasis’) and energy metabolism (e.g. ‘mitochondrial transport’ and ‘tricarboxylic acid cycle’), which may be associated with its salt resistance (Fig. 2c; Table S16, see online supplementary material). Some genes in the unique gene families of *P. pruinosa* were up-regulated under drought and/or salt stress, including *SK11* (shaggy-related kinase 11, PprTF14G0683.1), which is a regulator of Glc-6-phosphate dehydrogenase activity and important for acclimation to salt stress [30]; the *OASA1* (O-acetylserine (thiol) lyase (OAS-TL) isoform A1, PprTF05G0317.1 and PprTF05G0318.1), which originated from tandem duplication and can enhance the salt tolerance of plants through a regulatory pathway mediated by the hormone ABA [31].

**Figure 4:**
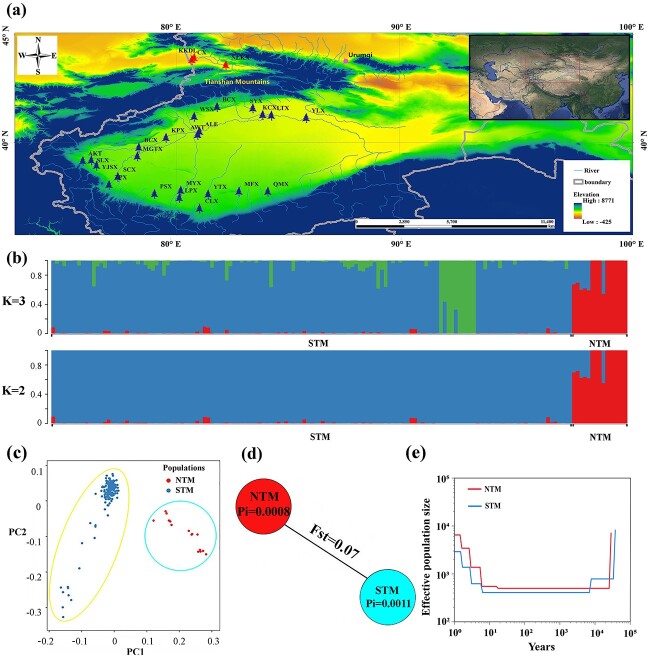
Population structure of *Populus pruinosa*. (**a**) Geographical distribution of 156 *P. pruinosa* accessions from 25 populations. Blue, and red tree tags on the Xinjiang, China map represented *P. pruinosa* populations of southern and northern Tianshan, respectively. (**b**) Population structure of 156 resequencing accessions. ‘NTM’, northern group of Tianshan Mountains. ‘STM’, southern group of Tianshan Mountains. (**c**) PCA analysis of *P. pruinosa* accessions. (**d**) Genetic diversity (Pi) and divergence between NTM and STM. (**e**) The assessment of effective population size (*N_e_*).

Gene family expansion (104) related to ‘defense response to virus’, ‘response to wounding’, and ‘photosynthesis’ in desert poplars’ (*P. pruinosa* and *P. euphratica*) ancestor facilitated their adaptation to cope with harsh desert environments (Fig. S7c and Table S17, see online supplementary material). Post divergence, *P. euphratica* (823) amplified temperature response (e.g. ‘response to freezing’, and ‘cellular response to heat’) and antioxidant capacity (e.g. ‘response to reactive oxygen species’ and ‘response to oxidative stress’) through further gene family expansion (Fig. S7d and Table S18, see online supplementary material), while *P. pruinosa* (386) boosted ion transport (e.g. ‘calcium-mediated signaling’, ‘potassium ion transport’, and ‘phosphate ion transmembrane transport’) and energy metabolism (e.g. ‘photosynthesis, light reaction’ and ‘glyoxylate cycle’) capabilities (Fig. 2d; Table S19, see online supplementary material). Notably, some genes in the expanded gene families of *P. pruinosa* were up-regulated under salt and/or drought stress, including *KUP6* (K^+^ uptake permease 6, PprTF08G1343.1), which acts as a key factor in K^+^ homeostasis under osmotic adjustment responses [32]; *ICL* (isocitrate lyase, PprTF07G1019.1 and PprTF17G0180.1), which participated in the regulation of salt tolerance in plants [33]; *CPK30* (calcium-dependent protein kinase 30, PprTF001Sca0018.1, PprTF15G0552.1, and PprTF12G0182.1), whose overexpression can increase the resistance of plants to salinisation [34]; and *GSTF6* (PprTF02G1860.1 and PprTF02G1859.1), originated from tandem duplication, was also found in an expanded gene family. These expanded gene families might be important for *P. pruinosa* to survive in highly saline environments. In addition, after differentiating from *P. euphratica*, 22 genes appeared to undergo specific positive selection in the *P. pruinosa* (Fig. 3b; Table S20, see online supplementary material), including *PSUT* (plastidic sugar transporter, PprTF01G2055.1), whose variants affected plant freezing tolerance and inflorescence development [35].

### Population structure analysis of *P. pruinosa*

The WGS was conducted on 156 individuals from 25 populations across *P. pruinosa*’s distribution range in China (Fig. 4a; Tables S21–S22, see online supplementary material). The data generated 1 591 438 SNPs and 189 491 indels (Table S23, see online supplementary material). A total of 1 255 987 and 99 735 SNPs were distributed in intergenic and genic regions, respectively, and were used for subsequent population-based analyses.

The evolutionary history of *P. pruinosa* in China was investigated by assessing the Delta K (individual ancestry coefficients) values according to the identified SNPs. Two groups (K = 2) represented the best model and revealed two distinct clades [southern group of Tianshan Mountain (STM) and northern group of Tianshan Mountain (NTM)] (Fig. S8a, see online supplementary material). The STM consisted of *P. pruinosa* specimens collected from southern Xinjiang, forming the basal clade, while the NTM included specimens from northern Xinjiang (Fig. 4b). Principal component analysis (PCA) and phylogenetic analysis further supported the two genetic clades in structure analysis (Fig. 4c;Fig. S8b, see online supplementary material). Pairwise population fixation statistics (Fst) between populations indicated the moderate genetic divergence between STM and NTM (Fst = 0.07) (Fig. 4d). Nucleotide diversity (Pi) was higher in the STM (Pi = 0.0011) than in the NTM (Pi = 0.0008), indicating the higher diversification of *P. pruinosa* in southern Xinjiang, China. Moreover, the STM showed a faster linkage disequilibrium (LD) decay, providing further evidence of higher genetic diversity in southern Xinjiang, China (Fig. S9, see online supplementary material).

The SMC++ was employed with WGS data to assess the effective population size (*N_e_*) of *P. pruinosa* (Fig. 4e). The decrease in *N_e_* of *P. pruinosa* was found to be associated with the last glacial maximum (~20 kya). The *P. pruinosa* population then entered a bottleneck period. After this bottleneck, the *N_e_* showed a gradual increase until the current maximum value because of global desertification control initiatives and ecological protection [36, 37].

### Identification of candidate loci for environmental adaptation

**Figure 5:**
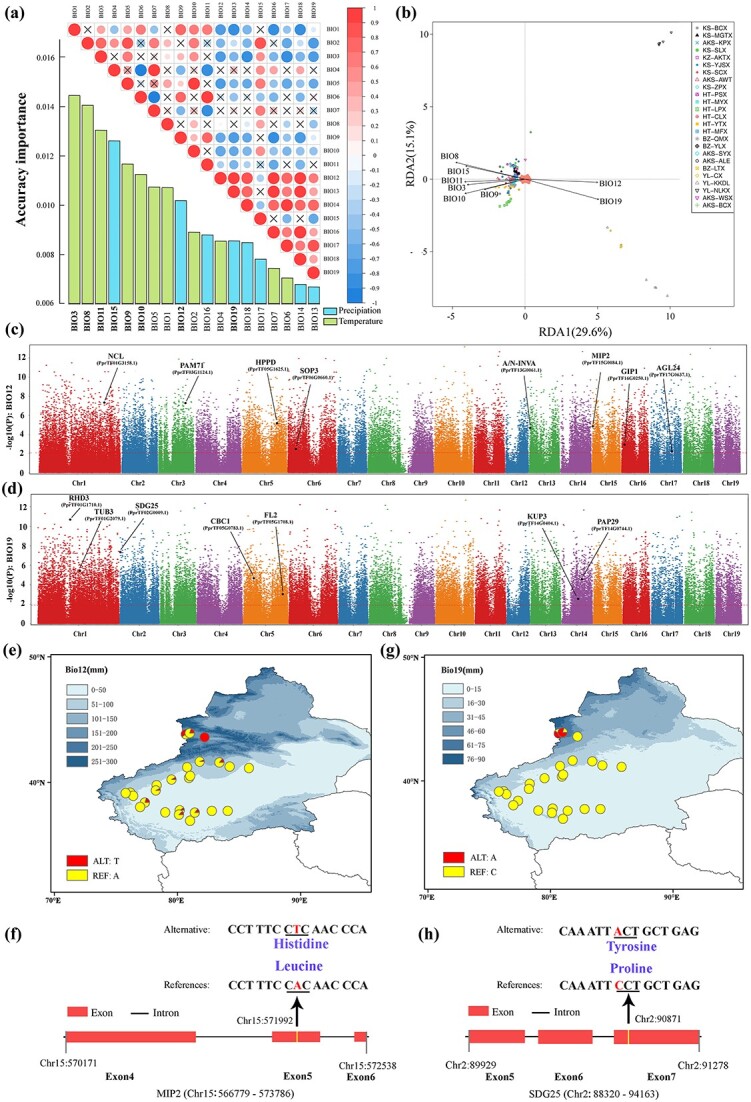
Identification of candidate loci for local adaptation. (**a**) Spearman’s correlation (above the diagonal) and gradient forest analysis ranking (below the diagonal) of 19 environmental variables. The bold variables indicate the six environmental variables selected for RDA analysis. (**b**) PCA plot based on RDA axes 1 and 2. (**c**) and (**d**) represented Manhattan plots of variants associated with annual precipitation (BIO12) and coldest season precipitation (BIO19), respectively. Dashed horizontal lines represent significance thresholds (*P* = 0.05). (**e**), (**g**) Allele frequencies of candidate adaptive SNPs (**e**, Chr15:571990; **g**, Chr2:90871) associated with BIO12 (**e**) and BIO19 (**g**) across the 25 populations. (**f**), (**h**) Alleles of candidate adaptive SNPs alter protein sequences in exon regions *MIP2* (**f**) and *SDG25* (**h**).

To detect genetic variants associated with environmental factors, two complementary methods of genotype-environment association (GEA) were utilized. Firstly, the latent factor mixed model (LFMM) was utilized to test for GEA across 19 temperature- and precipitation-related variables (Table S24, see online supplementary material). A total of 55 750 SNPs associated with environmental variables were identified. (Table S25, see online supplementary material). Considering the multicollinearity and the priority of the variables, a redundancy analysis (RDA) was performed using five temperature variables [isothermality (BIO3), mean temperature of wettest quarter (BIO8), mean temperature of driest quarter (BIO9), mean temperature of warmest quarter (BIO10), and mean temperature of coldest quarter (BIO11)] and three precipitation variables [annual precipitation (BIO12), precipitation seasonality (BIO15), and precipitation of coldest quarter (BIO19)] (Table S26, see online supplementary material). These variables were chosen based on their ranked importance obtained through gradient forest analysis and their correlations with each other (Fig. 5a), with a Spearman correlation coefficient of |r| < 0.75. The first three out of the six RDA axes exhibited statistical significance, accounting for approximately 55.24% of the variance captured by the RDA model (Figs. 5b; Fig. S10, see online supplementary material). Furthermore, of the variants identified by LFMM, 3 799 SNPs (1 433 genes) displayed pronounced loadings (standard deviation >3) along at least one RDA axes (Table S27, see online supplementary material). These shared variants, known as ‘core adaptive variants’, were crucial for local climate adaptation, mostly association with ‘petal development’, ‘regulation of photoperiodism, flowering’, ‘malate metabolic process’, ‘cellular amino acid metabolic process’, ‘sugar mediated signaling pathway’ and ‘tricarboxylic acid cycle’. (Fig. S11, see online supplementary material). Additionally, a higher number of adaptive variants were found to be linked to precipitation-related variables (Fig. S12, see online supplementary material).

Owing to the precipitation difference between the STM (annual precipitation <55 mm) and NTM (annual precipitation >200 mm), precipitation-responsive genes possibly affect adaptation. For example, *MIP2* (MAG2-interacting protein 2) variants were strongly associated with BIO12 (Fig. 5c). *MIP2* plays an integral role in modulating seed viability, ABA stress, and salt tolerance [38]. A candidate adaptive SNP situated within an exon region (Chr15:571992) of *MIP2* (PprTF15G0084.1) was selected to display the geographic distribution of allele frequencies. The T allele predominated in the northern region with heavy annual precipitation, while the A allele was nearly fixed in low rainfall areas (Fig. 5e; Table S28, see online supplementary material). We then evaluated the effect of base mutations on the coding region of *MIP2* and found that the transition of the A allele to the T allele led to nonsynonymous mutations in amino acids (Fig. 5f), that is, the transition between leucine (hydrophobic amino acid) and histidine (hydrophilic amino acid).

The winter drought can delay flowering and even cause damage to woody plants [39–42]. There is a difference in the precipitation of coldest quarter between STM (<4 mm) and NTM (>35 mm). Here, we found that *SDG25* (SET domain protein 25) variants were strongly associated with BIO19 (Fig. 5d). *SDG25* is a loss-of-function ortholog mutant and has an early flowering phenotype [43]. A candidate adaptive SNP situated within an exon region (Chr2:90871) of *SDG25* (PprTF02G0009.1) was selected to display the geographic distribution of allele frequencies. The C allele predominated in the northern region with heavy precipitation in the coldest quarter, while the A allele was nearly fixed in low rainfall areas in the coldest quarter (Fig. 5g; Table S28, see online supplementary material). The effect of base mutations on the coding region of *SDG25* was evaluated. The transition of the C allele to the A allele led to nonsynonymous mutations in amino acids (Fig. 5h), that is, the transition between proline (hydrophobic amino acid) and tyrosine (hydrophilic amino acid). These results suggested that variation of *MIP2* and *SDG25* were the results of the adaptive evolution of *P. pruinosa* in response to different precipitation zones in desert environments.

## Discussion

The chromosome-level genome of *P. pruinosa* lays a foundation for investigating the genetic and evolutionary mechanisms of its adaptation to extreme saline-alkali desert environments. In this study, we assembled a 521.09 Mb chromosome-scale genome of *P. pruinosa* (v2.0) *de novo*. Compared with the *P. pruinosa* genome (v1.0) based on short reads [11], our assembly achieved higher degrees of contiguity and completeness in a variety of aspects. This study will contribute to further investigation of the genomic basis of distinctive traits and environmental adaptation of *P. pruinosa*. For example, high-quality genome can facilitate the identification of complete gene duplication events [17, 18]. The genes originating from WGD laid an adaptive foundation for the survival (‘response to water deprivation’) and reproduction (‘flower development’) of desert poplars (*P. pruinosa* and *P. euphratica*) in harsh environments. The tandemly duplicated genes (e.g., *RCI2A* [22, 23], *ERD4* [24], and *GSTF6* [20, 21]) of the *P. pruinosa* genome were mainly associated with stress responses, which might enhance the ability of *P. pruinosa* to cope with complex desert environments (especially high salinity and drought). Furthermore, the availability of the chromosome-level *P. pruinosa* genome would facilitate comprehensive investigation into the distribution of repetitive elements and their potential impact on *P. pruinosa* evolution. Here, the peak around 0.45 Mya of the LTR-RT burst in the *P. pruinosa* genome appeared earlier than that in *P. euphratica* (0.40 Mya) and *P. trichocarpa* (0.35 Mya), particularly appearing when the quaternary climates were considerably oscillating. However, the underlying mechanism driving the LTR-RT burst requires further comprehensive and detailed investigation. Although the occurrence of these repetitive elements is biased towards gene-poor chromosomal regions, LTR-RT inserted genes exhibit a large difference in expression levels and high protein evolutionary rates than the non-LTR-RT inserted genes between the orthologous genes in *P. pruinosa* and *P. euphratica*, indicating that the recent amplification of repeats might contribute to the genomic adaptive evolution and the species differentiation between desert poplars.

More gene families involved in salt tolerance emerged in *P. pruinosa* but not in its sister, *P. euphratica*. Although *P. pruinosa* and *P. euphratica* were both relict plants and had a close relationship, *P. pruinosa* exhibited stronger salt tolerance [6,7]. *SK11* [30] and *OASA1* [31] were found in the unique gene families of *P. pruinosa*, and *GSTF6* [20, 21] *KUP6* [32], *ICL* [33], and *CPK30* [34] were found in the expanded gene families of the *P. pruinosa* genome by comparative genomic analysis. Moreover, these genes were co-expressed in *P. pruinosa* under salt stress, further supporting the tolerance of *P. pruinosa* to high-salt desert environments.

The ‘core adaptive variants’ of the *P. pruinosa* population to temperature and precipitation in China were identified by LFMM and RDA analysis. Environmental heterogeneity can drive genetic differentiation and local adaptation in plants [44]. Given the high mountain barrier created by the Tianshan Mountains in the middle of Xinjiang, *P. pruinosa* in China can be distinguished into two clades: STM and NTM. Previous study reported that different climates determined genomic diversity of *Populus koreana* in different directions, leading to its adaptation to local environments [45]. *P. pruinosa* in the NTM is exposed to high precipitation compared with the STM. Our results suggested that diverse pathways were involved in natural selection, such as ‘petal development’, ‘regulation of photoperiodism, flowering’, ‘malate metabolic process’, ‘cellular amino acid metabolic process’, ‘sugar mediated signaling pathway’ and ‘tricarboxylic acid cycle’. Therefore, we speculated that to effectively cope with the different climatic environments in the northern and southern of the Tianshan Mountain, *P. pruinosa* regulated the adaptive selection of relevant genes and balances adaptation and growth. We found several candidate loci associated with precipitation-adaptive traits, including *MIP2* and *SDG25. MIP2* associated with BIO12 is involved in ABA or salt stress response [38], and *SDG25* associated with BIO19 is involved in flowering [43]. Nonsynonymous mutations were observed in the exon region, and the geographic distribution of the allele frequencies showed population differentiation. These associated pathways and genes indicated that multiple pathways were involved in the local adaptation of *P. pruinosa* and in polygenic adaptation. In future, investigation on these genes’ functions and the assembly of *P. pruinosa* haploid genome, as well as the construction of pan-genome, are necessary to further exploit the rich genetic variations and illuminate the adaptive mechanism of desert poplars to the saline-alkaline desert ecosystem. Our research provides a genome-level perspective for the genetic evolution and ecological adaptation of *P. pruinosa*, laying a foundation for the genetic breeding of *P. pruinosa*, which is conducive to the recovery and reconstruction of desert ecosystems.

## Materials and methods

### Samples used for genome sequencing

The *P. pruinosa* samples utilized in this study were gathered from Aral, Xinjiang Uygur Autonomous Regions, China. For genomic sequencing, genomic DNA of young leaves from one female *P. pruinosa* (‘XX’, XY sex-determination system [46]) was extracted using a DNAsecure plant kit (TIANGEN Biotech, Beijing, China). A sequence library with an insert size of approximately 350 bp was constructed, resulting in the production of 30.7 Gb raw reads through Illumina PE150 sequencing. In addition, SMRTbell library with a length of 20 kb was prepared and sequenced using PacBio Sequel II, and consensus reads (HiFi reads) were generated using CCS software (https://github.com/pacificbiosciences/ccs) with default parameters (Table S2, see online supplementary material).

We extracted DNA from the juvenile floral buds of the same *P. pruinosa* to construct the Hi-C library according to the standard protocol described previously with some modifications [47]. After the disintegration of bud cells, the extracted chromatin was cross-linked with formaldehyde and digested with the 4-cutter restriction enzyme DpnII. The purified DNA, fragmented into 300–500 bp fragments using protease, was ligated to sequencing adaptors. The biotin-labeled fragments were collected utilizing streptavidin C1 magnetic beads. The PCR-enriched libraries (12–14 cycles) were sequenced using Illumina PE150, generating approximately 72.8 Gb of raw data.

The total RNA of shoots, fruit, and three distinct leaf morphologies (oblong, round, and broad-ovate leaves) from *P. pruinosa* was extracted using an RNAprep pure plant kit (Tiangen Biotech). Subsequently, a cDNA library was prepared employing the NEBNext Ultra RNA library prep kit for Illumina (New England Biolabs), followed by sequencing on an Illumina NovaSeq 6000 platform. All sequencing procedures were performed by Novogene Co., Ltd (Beijing, China).

### Estimation of genome size and genome assembly

The k-mer statistic was used in estimating the size of the *P. pruinosa* genome with a k-mer-based method [48]. HiFi reads were assembled de novo with Hifiasm v0.13 [49] with default parameters: ‘-N 100 -r 3 -x 0.8 -y 0.2 -s 0.75’. Then, based on the Hi-C sequencing data, contig sequences were anchored onto 19 chromosomes by using ALLHIC v0.9.8 [50] with the following parameters: ‘K 19 --minREs 50 --maxlinkdensity 3 --NonInformativeRabio 0’ and Juicerbox v1.11.08 [51]. The Illumina DNA sequencing libraries were mapped to the genome assembly using BWA v0.7.15 [52].The LTR_retriever v2.9.0 [53] was employed for precise identification of LTR-RTs, while the LAI was computed using the default parameters of LTR_Finder v1.0.6 [54]. The completeness of genome assembly and prediction of protein-coding genes were evaluated by BUSCO (odb10) [55]. The QV of genome assembly was assessed using Merqury V1.3 [56], which is based on 21-mer database from Illumina short reads.

### Repeat annotation

The TEs of the *P. pruinosa* genome were identified through a combination of *de novo* and homology-based methods. The homolog prediction commonly used Repbase database employing RepeatMasker v4.1.0 [57] software and its in-house scripts (RepeatProteinMask v4.1.0) with default parameters to extracted repeat regions. Additionally, *de novo* repetitive elements were predicted using LTR_Finder v1.0.6, RepeatScout v1.0.5, and RepeatModeler v2.0.1 with default parameters. The raw TE library consisted of repeat sequences longer than 100 bp with less than 5% gap ‘N’. To identify repetitive sequences at DNA-level, a custom library was employed in RepeatMasker. SSR was identified using the Misa software v2.1 according to a previously described method [58], with the following definition: ‘1-10 2-5 3-4 4-3 5-3 6-3, interruptions 0’.

### Annotation of genome sequences

The RNA-seq data obtained from three distinct tissues (shoots, fruits, and leaves) were utilized to facilitate the annotation of gene structures (Table S29, see online supplementary material). A strategy that combined *ab initio* strategy and homology-based genes prediction was employed (Table S30, see online supplementary material). The protein sequences of five Salicaceae plant genomes, including *Salix purpurea* [59], *Salix suchowensis* [60], *P. trichocarpa* [61], *Populus alba* [62], and *P. euphratica* were downloaded. The TBLASTN v2.2.26 [63] (E-value ≤1e−5) was employed to align the protein sequences to the genome. Subsequently, GeneWise v2.4.1 [64] was used for accurate spliced alignments and gene structure prediction within each protein region. The Augustus v3.2.3 [65], Geneid v1.4 [66], Genescan v1.0 [67], GlimmerHMM v3.04 [68], and SNAP [69] were used by automated gene prediction pipeline. The Trinity was used to assemble RNA-seq sequences and then Hisat v2.0.4 [70] and TopHat v2.0.11 [71] with default parameters were used to align RNA-seq data from different tissues to the genome and identify exonic regions and splicing position. The results were used as inputs for Stringtie v1.3.3 [72] and Cufflinks v2.2.1 [73] with default parameters for genome-based transcript assembly. All predicted gene models were integrated using EvidenceModeler (EVM) v1.1.142 [74] and filtered with PASA [75] for the generation of non-redundant gene models.

The functional annotation of protein-coding genes is performed through comparing them to the NCBI non-redundant proteins [76], SwissProt [77], and InterPro databases using BLASTP v2.2.26 [78], with a maximum E value of 1e−5. Domains were annotated by searching against the Pfam [79] database with HMMER v3.0 [80]. GO terms and Kyoto Encyclopedia of Genes and Genomes pathways were used in identifying their best functional classification. The tRNA and miRNA were identified by searching against the Rfam database v14.1 [81] by using INFER-NAL [82]. The snRNA fragments were predicted using tRNAscan-SE v1.4 [83]. The rRNA sequences were predicted by alignment to the rRNA sequences of related species by using BLASTN with an E-value <1e−10.

### Genomic evolutionary analysis

Based on AEK [16], the evolutionary route of the genes was constructed for *P. pruinosa* and the other four species (*V. vinifera*, *A. thaliana*, *P. trichocarpa*, and *P. euphratica*) in MCScanX. The analytical method of LTR-RTs in the *P. pruinosa* genome was described in the repeat annotation section. The gene duplication type in the genomes of *P. pruinosa* and *P. euphratica* were identified using the duplicate_gene_classifier script in MCScanX [84].The initial identification of LTR-RTs was performed using LTR_Finder v1.0.6 [50] (with parameters: ‘-D 20000 -d 1000 -M 0.85’) and LTRharvest v1.5.10 [85] (with parameters: ‘-similar 85 -motifmis 1’). The identification and calculation of insertion time for high-quality intact LTR-RTs were accomplished using LTR_retriever v2.9.0 with default parameters [53]. The neutral mutation rate of 2.5 × 10^−9^ mutations per bp per year was used [61]. The nonsynonymous (Ka) and synonymous (Ks) substitution rates of homologous gene pairs between *P. pruinosa* and *P. euphratica* [86] were calculated by PAML v4.9 [87]. GO enrichment of genes were performed using the R package clusterProfiler [88].

### Gene expression analysis

We evaluated the influence of LTR-RTs insertion on gene expression in *P. pruinosa* by aligning RNA-seq data from normally developing leaves of both *P. pruinosa* and *P. euphratica* to their respective reference genomes using HISAT2 [89]. The quantification of gene expression levels was performed using StringTie v1.3.4d [90], while orthologs were identified through reciprocal best hit analysis with BLASTP v2.2.26 [78]. Finally, we calculated the fold change (FC) in gene expression level for putative orthologs between *P. pruinosa* and *P. euphratica*.

To explore the molecular events of *P. pruinosa* under drought and salt stress, we collected 3-day germination seedlings treated with 15% PEG 6000 and 0.3 mol/L NaCl, respectively, and untreated (without PEG 6000/NaCl) for transcriptome analysis. RNA extraction, identification and quantitative analysis of reads were carried out by Frasergen Co., Ltd (Wuhan, China). Genes with |log2FC| > 1 and *P*-value <0.05 were treated as differentially expressed genes using DESeq2 (v1.10.1). Detailed information can be found in File S1 (see online supplementary material).

### Gene family and phylogenomic analysis

We used Orthofinder v2.4.0 [91] with default parameters to identify homologous gene cluster sources for *P. pruinosa* and the other 13 genome datasets listed in Table S31, see online supplementary material). These sources included eight Salicaceae species (*P. deltoides*, *P. tremula*, *P. euphratica*, *P. trichocarpa*, *Salix brachista*, *Salix viminalis*, *S. purpurea*, and *S. suchowensis*), two Euphorbiaceae species (*Ricinus communis* and *Manihot esculenta*), one Celastraceae species (*Tripterygium wilfordii*), one Brassicaceae species (*A. thaliana*) and one Vitaceae species (*V. vinifera*). A maximum-likelihood tree (*V. vinifera* as an outgroup) was constructed according to the amino acid sequences of single-copy orthologous genes shared by 14 species by using IQ-TREE v1.6.11 [92] with the GTR + F + I + G4 model. We estimated divergence times using MCMCtree in the PAML v4.0 package [93]. The approximate likelihood method with correlated substitution rate (clock = 3) was used. Gradient and Hessianl matrix were employed, and samples were drawn every 20 iterations up to 5 000 000 iterations. The analysis consisted of 100 000 000 iterations, and the first 100 000 were discarded as burn-in. To ensure convergence to the stationary distribution, we conducted each analysis (including the CodeML step) in duplicate and compared the results between runs. Five calibration points were applied based on the TimeTree database (http://timetree.org/) and a recent study for plastome phylogeny of Salicaceae: *A. thaliana* and *V. vinifera* origination time (107–135 Mya), *A. thaliana* and *T. wilfordii* origination time (88–101 Mya), *M. esculenta* and *R. communis* origination time (58–85 Mya), Salicaceae origination time (48–52 Mya) [94,95], and *Populus* origination time (6.6–11.3 Mya) [96].

The orthologous genes and phylogenetic tree topology of 14 species were analysed using Orthofinder. The resulting data was input into CAFE v.4.2.1 [97] with default parameters. CAFE employed a random birth and death model to estimate the size of gene families at each ancestral node. Significance of expansion or contraction was determined using a *P*-value cutoff of 0.05, and then we conducted a positive selection analysis (ω > 1, *P* < 0.05) based on the single-copy orthologous genes of 14 species using the branch-site model of the PAML v4.0 package [93].

### Whole-genome resequencing and SNP calling

A total of 156 individuals (more than 50 m between individuals) were collected from 25 natural populations of *P. 27ruinose* in Xinjiang, China, covering the entire distribution range of the species in China. The genomes of the 156 individuals were resequenced using a 150 bp paired-end read on the Illumina HiSeq 2500 platform. All raw reads were filtered by Fastp v0.23.0 [98], which was utilized to filter all raw reads, and BWA v0.7.15 [52] was employed to align the reliable clean reads to the reference genome of *P. pruinosa*. To eliminate PCR duplication, Samtools v1.3.1 [99] was applied. Then, GATK v3.7 [100] was used for SNP calling, and ANNOVAR [101] was used for variant annotation and the prediction of the effect of variants on gene function. Based on genotypic data, kinship coefficients among samples were calculated using KING v2.2.6 [102], revealing no relatedness (InfType: UN) among the 156 samples. In addition, 15 randomly selected SNP loci were tested by Sanger sequencing in five samples, respectively, for genotype verification. The accuracy of detection results was more than 95%, indicating that the resequencing results were reliable (Table S32, see online supplementary material).

### Population structure inference

The fastSTRUCTURE v2.3.4 [103] was used in determining the optimal number of subpopulations based on SNP variation among the sequenced 156 genotypes. PCA was carried out by PLINK v1.90p [104]. The neighbor-joining method in MEGA v7.0 [105] was used in constructing a phylogenetic tree of the *P. pruinosa* population. Through population structure analysis, phylogenetic analyses, and PCA analysis, we defined two groups of individuals: STM and NTM. LD decay for each group was estimated for all pairs of SNPs using PopLDdecay v3.4 [106] with the following parameters: ‘-MAF 0.05 -MaxDist 1000’. In addition, we used SMC++ v1.15 [107] to infer the population size histories of two populations based on unphased SNPs with MAF > 0.05.

### Identification of environment-associated genetic variants

We used a univariate LFMM implemented in the LEA v3.3 [108] R package to investigate the relationship between allele frequencies and environmental variables. Each environmental variable underwent five separate MCMC runs, with 5 000 iterations as burn-in followed by 10 000 iterations. The resulting *P*-values were averaged and adjusted for multiple tests (false discovery rate correction of 5% as the significance cutoff). Additionally, we employed RDA [109] to identify genetic variants that strongly correlated with multivariate environmental axes. To assess the relative importance of 19 environmental variables, GF analysis was performed through GradientForest [110] R package. Furthermore, for RDA analysis, we selected variables with pairwise correlation coefficients |r| < 0.75 through Vegan R package. Subsequently, we extracted the first three RDA model-constraint axes as significant environment-associated variants by applying a standard deviation cutoff of three along one or more RDA axes. The 3 799 adaptive variants identified through LFMM and RDA, referred to as ‘core adaptive variants’, were further annotated by ANNOVAR [101] for detailed investigation and comparison of their roles in the influence of the environment on shaping spatial genetic variation. This methodology has been described in previous reports [10, 45].

## Acknowledgements

We would like to thank all authors for their valuable discussions. This work was financially supported by the Natural Science Foundation of China (32371838 and U1303101), the Bingtuan Science and Technology Program (2021BB010), and the Postgraduate Research and Innovation Project of Tarim University (TDBSCX202003).

## Author contributions

J.H.S. performed investigation and writing-original draft. J.H.S. and J.D.X. performed formal analysis and visualization. C.Q., J.T.Z., S.H.Z., and X.Z. performed investigation. Z.H.W. performed project administration, acquisition of resources, and writing-review. Z.J.L. performed project administration, acquisition of resources, and funding acquisition. All authors revised and approved the final manuscript.

## Data availability

The raw sequence reads (including short reads, long reads, and Hi-C reads) for the *P. pruinosa* genome, as well as the RNA-seq under salt and drought stress were submitted to NCBI (National Center for Biotechnology Information) with the BioProject accession number PRJNA863418. The assembly and annotation files were available from figshare (https://figshare.com/articles/online_resource/Pprgenome_fa/20705107/2). The whole-genome sequencing data for 156 *P. pruinosa* accessions also were uploaded to NCBI under the accession number PRJNA865525.

## Conflict of interest statement

The authors declare no competing interests.

## Supplementary data

Supplementary data is available at *Horticulture Research* online.

## Supplementary Material

Web_Material_uhae034
