# Supplementary material for: The chromosome-scale genome and population genomics reveal the adaptative evolution of Populus pruinosa to desertification environment
Source: Hortic Res. 2024 Feb 6;11(3):uhae034. doi: 10.1093/hr/uhae034 (PMC10967694; doi:10.1093/hr/uhae034)
Supplement: Web_Material_uhae034 [file web_material_uhae034.zip › Supplemental File 1.docx]

**Gene expression analysis of *P. pruinosa* under drought and salt stress**

To explore the molecular events of *P. pruinosa* under drought and salt stress, we collected 3-day germination seedlings treated with 15% PEG 6000 and 0.3 mol/L NaCl, respectively, and untreated (without PEG 6000/NaCl) for transcriptome analysis. Seeds from Aksu Awat County (Xinjiang, China) were cultured under a photoperiod of 8 hours dark (25 °C) and 16 hours light (30 °C), with a photon flux density of 300 μM·m^−1^·s^−1^ in an artificial climate box. Total RNA was extracted from three biological replicates using Trizol reagent (Invitrogen, USA). RNA concentrations were measured using a Qubit 2.0 fluorometer (Life Technologies, USA). The NEBNext Ultra RNA library prep kit was used to prepare the cDNA library for sequencing. Sequencing was performed on the MGISEQ-2000RS platform. Low-quality reads containing adapters, unknown nucleotides (> 10% "N"), and those with a quality rating less than 50% (Q-value ≤ 20) were removed from the dataset. Hisat2 v2.1.087 was employed to map the clean reads to P. pruinosa genome (v2.0). Subsequently, Stringtie v1.3.488 was utilized for quantitative expression analysis of the genes in each sample. The FPKM value for each gene was calculated based on its length and the mapped read count. Genes with |log2FC| > 1 and p-value < 0.05 were treated as differentially expressed genes using DESeq2 (v1.10.1).
