# Supplementary material for: The chromosome-scale genome and population genomics reveal the adaptative evolution of Populus pruinosa to desertification environment
Source: Hortic Res. 2024 Feb 6;11(3):uhae034. doi: 10.1093/hr/uhae034 (PMC10967694; doi:10.1093/hr/uhae034)
Supplement: Web_Material_uhae034 [file web_material_uhae034.zip › Supplementary Figures.docx]

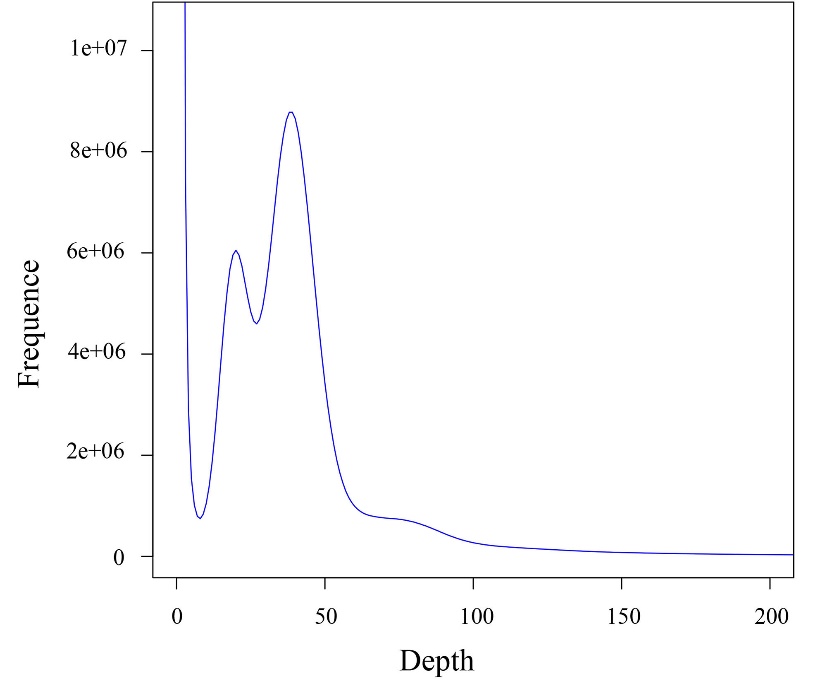


Figure S1. K-mer frequency distributions.


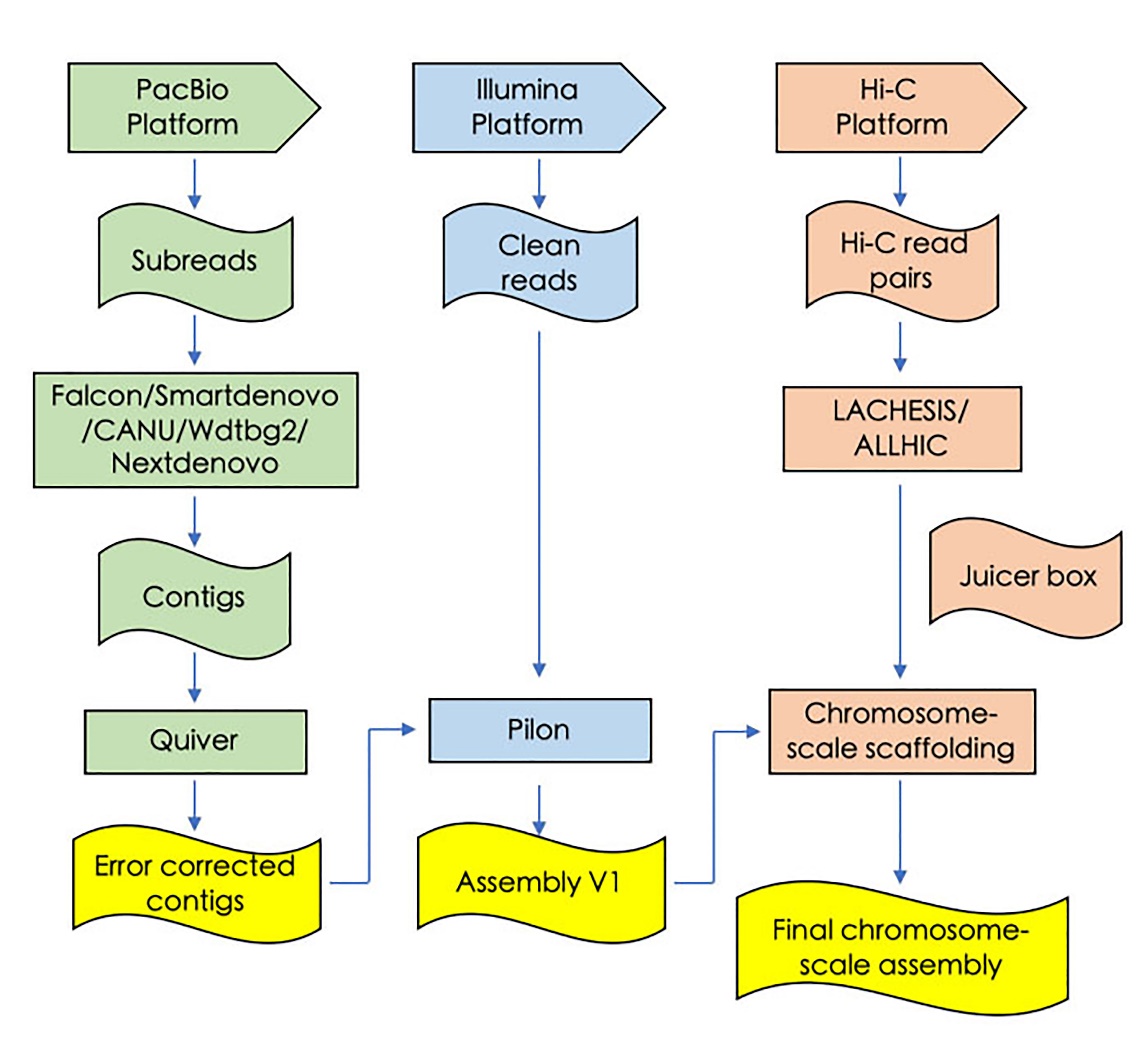


Figure S2. Overview of the processing pipeline used to assemble the *P. pruinosa* genome.


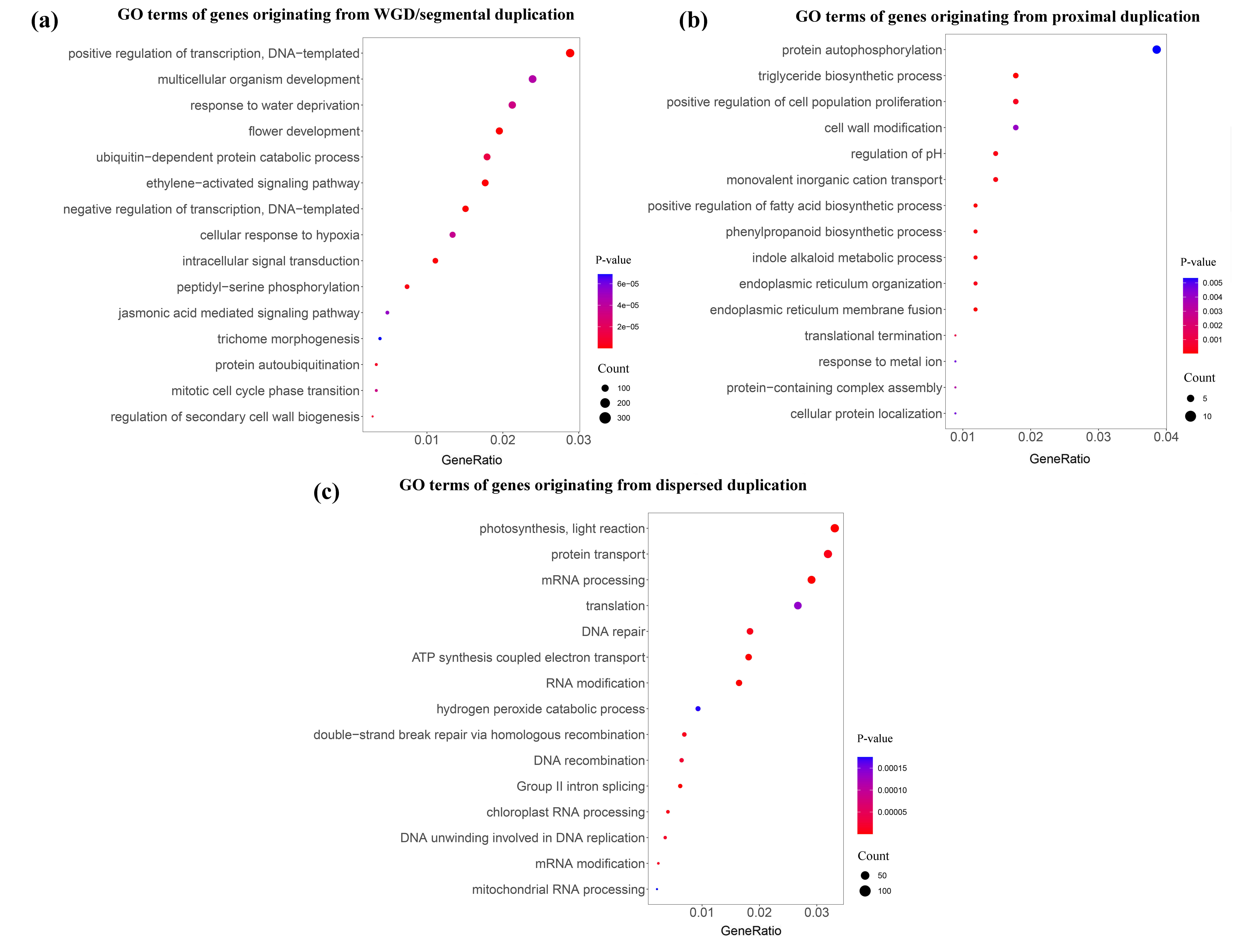
Figure S3. GO enrichment analysis for different gene duplication types in *P. pruinosa*.


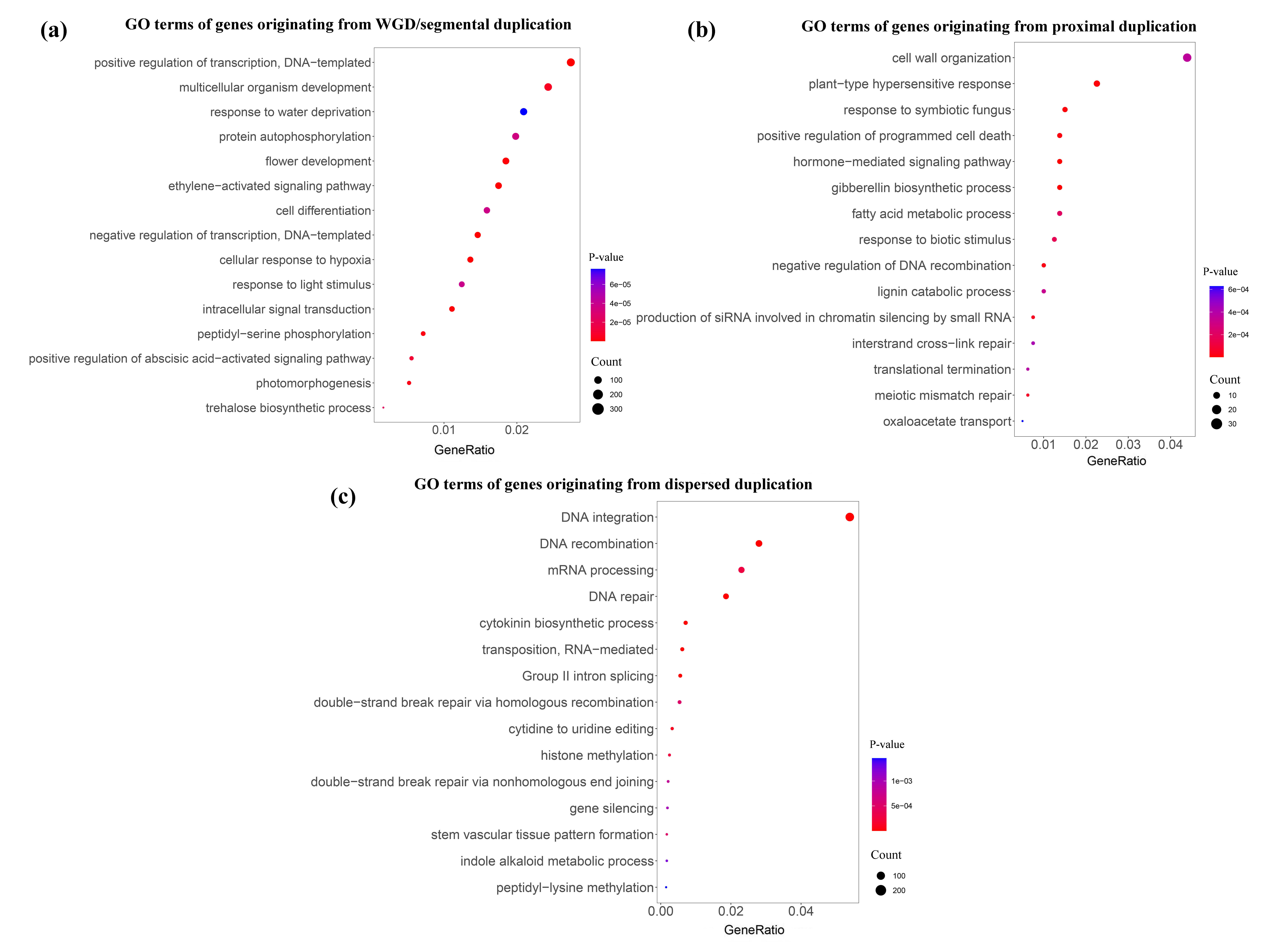
Figure S4. GO enrichment analysis for different gene duplication types in *P. euphratica.*


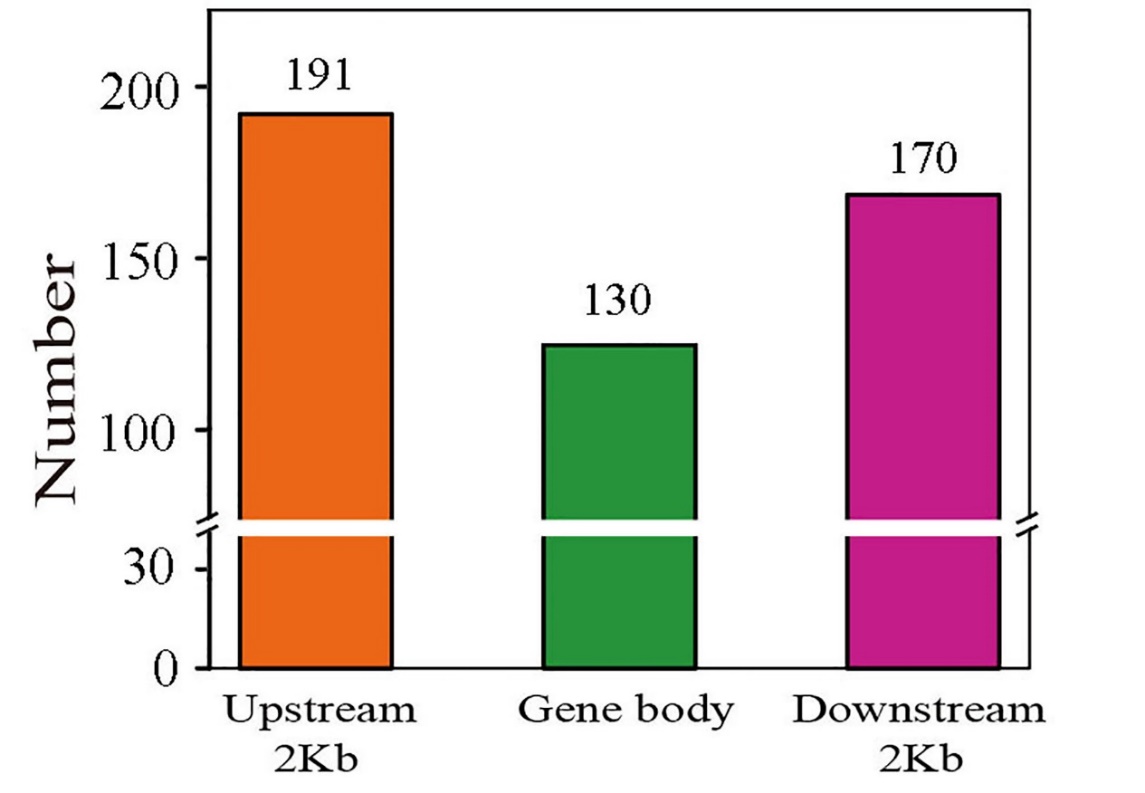


Figure S5. The number of LTR-RTs inserted into the three gene regions.


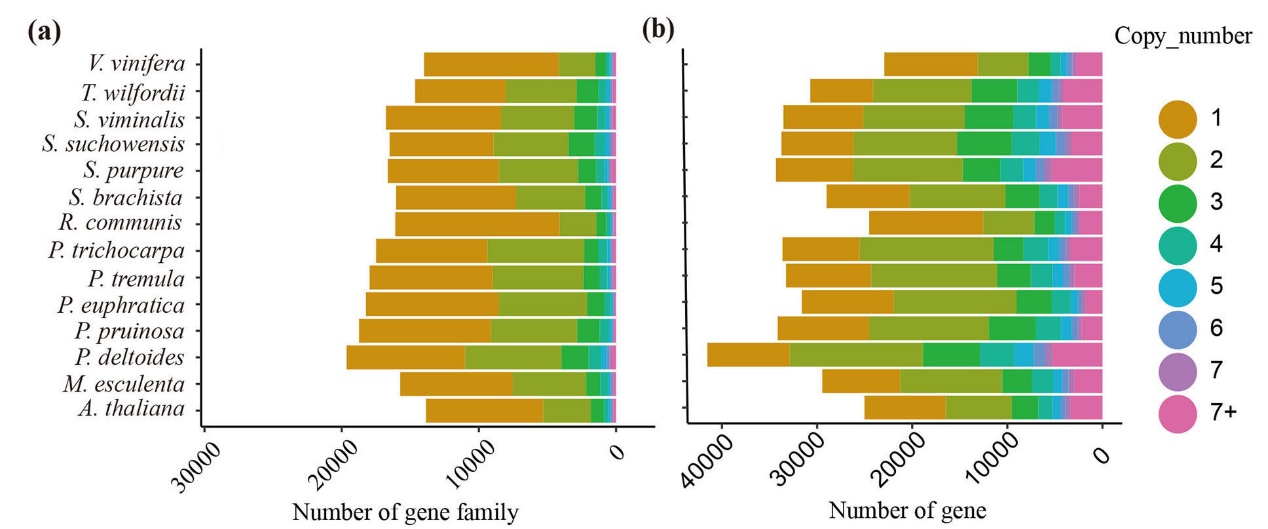


Figure S6. Gene family identification of 14 species genomes.


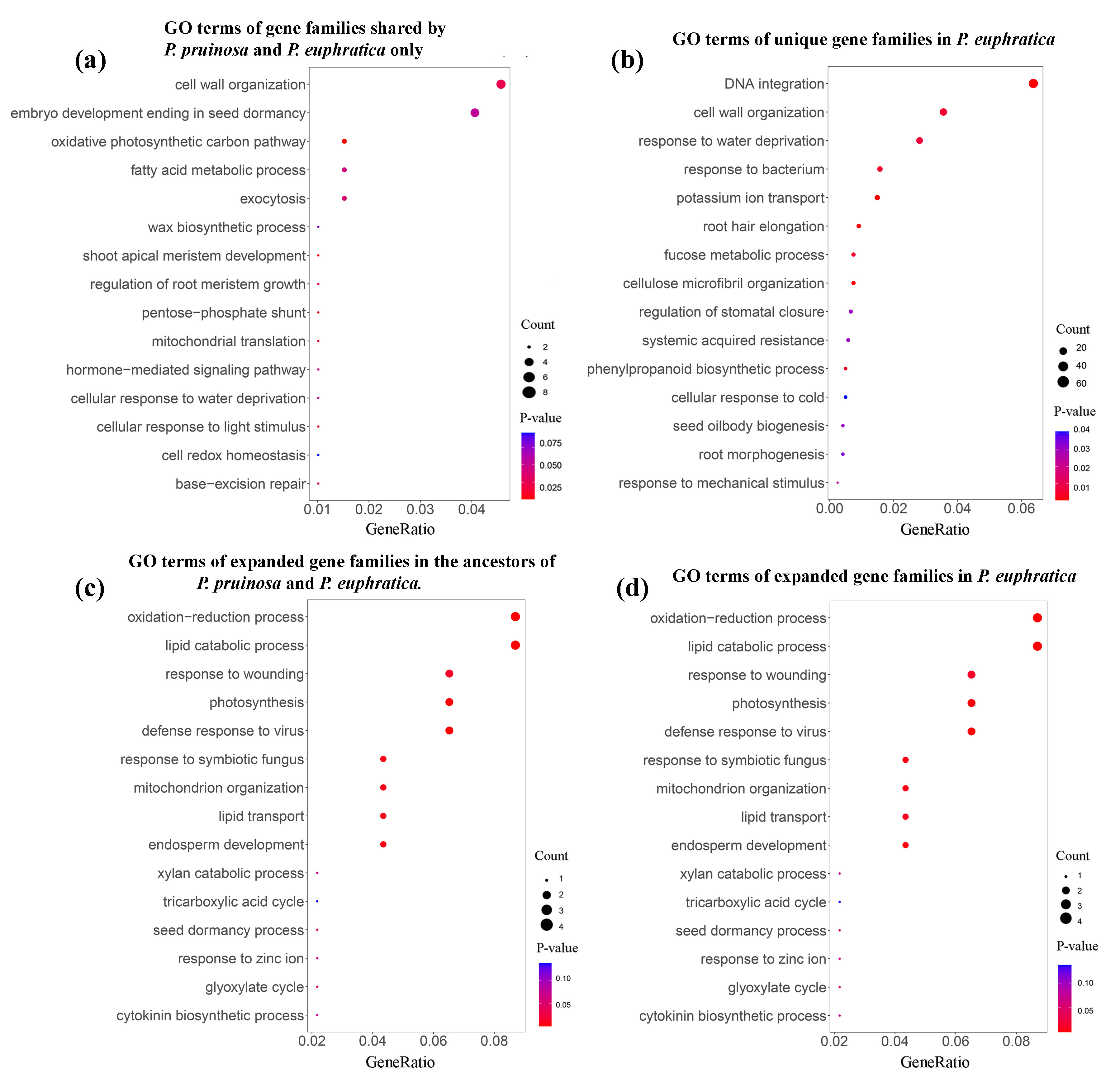
Figure S7. Gene family GO terms in comparative genome analysis of *P. pruinosa* and *P. euphratica*.


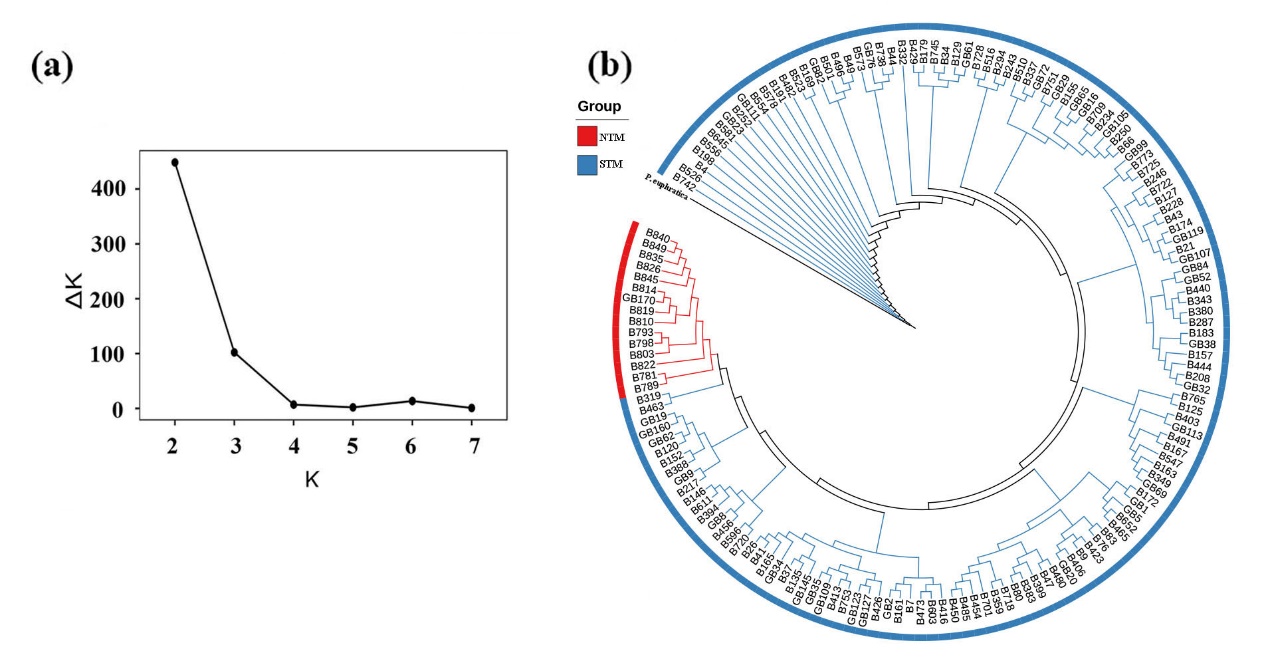


Figure S8. K value estimation and Neighbor-joining phylogenetic tree of *P. pruinosa* accessions.­


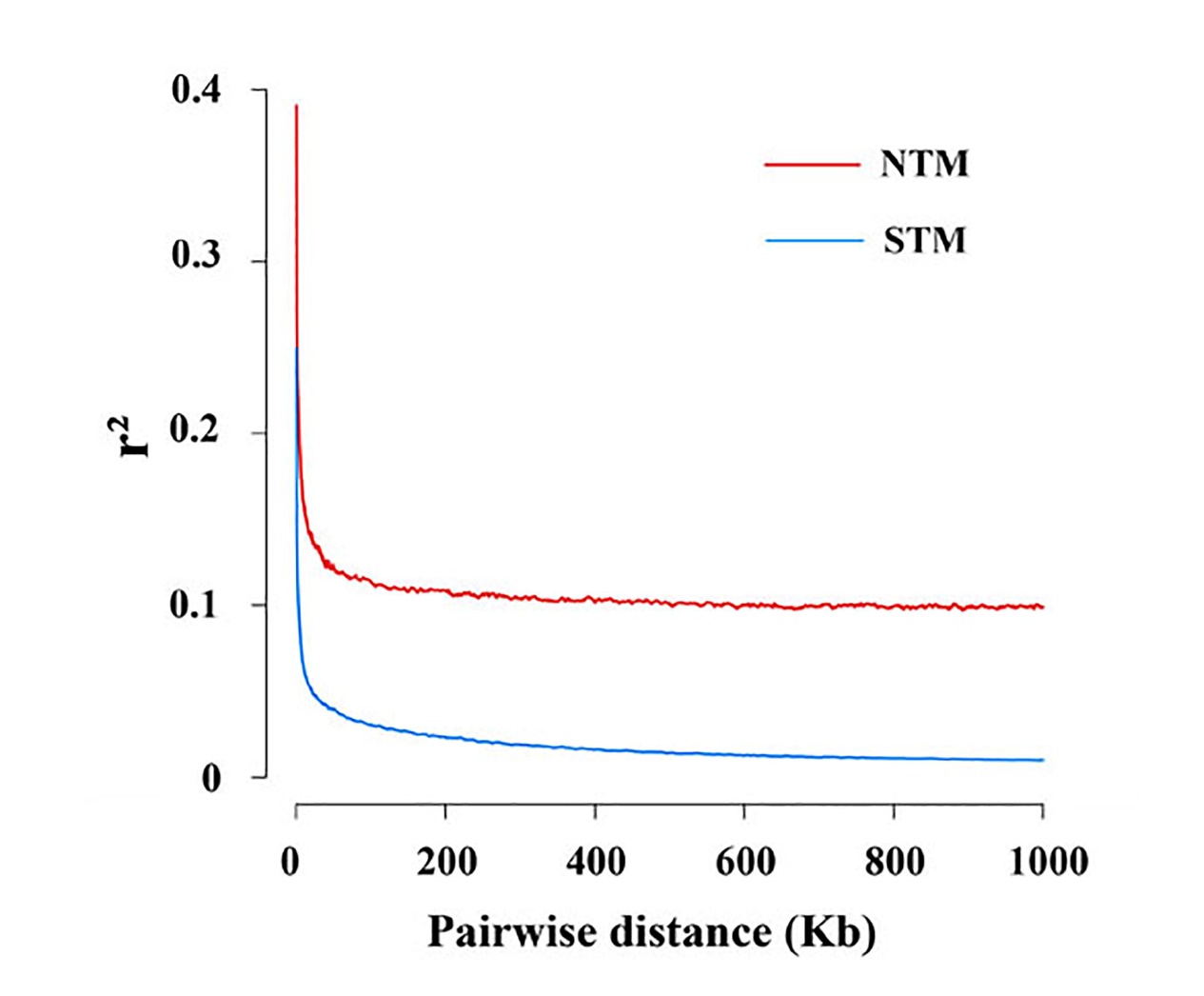


Figure S9. LD decay for the southern group of Tianshan Mountains and the northern group of Tianshan Mountains of *P. pruinosa* populations.


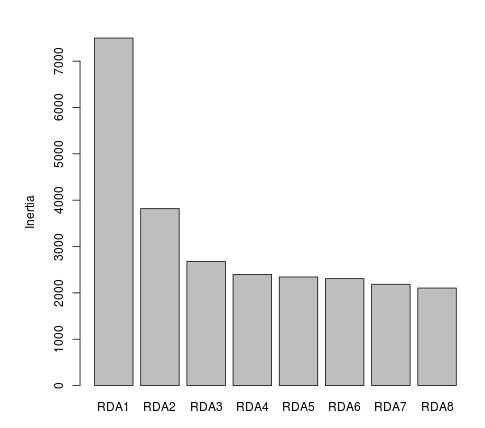


Figure S10. Variance explained by RDA axes.


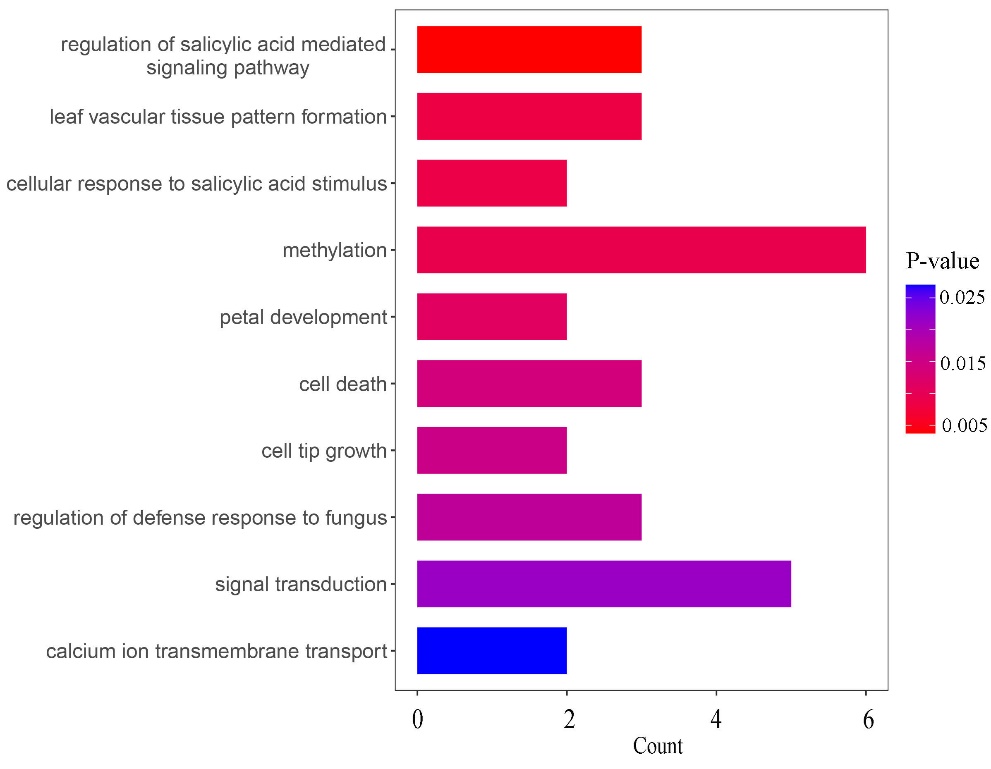


Figure S11. GO enrichment analysis of genes with core adaptive variants.


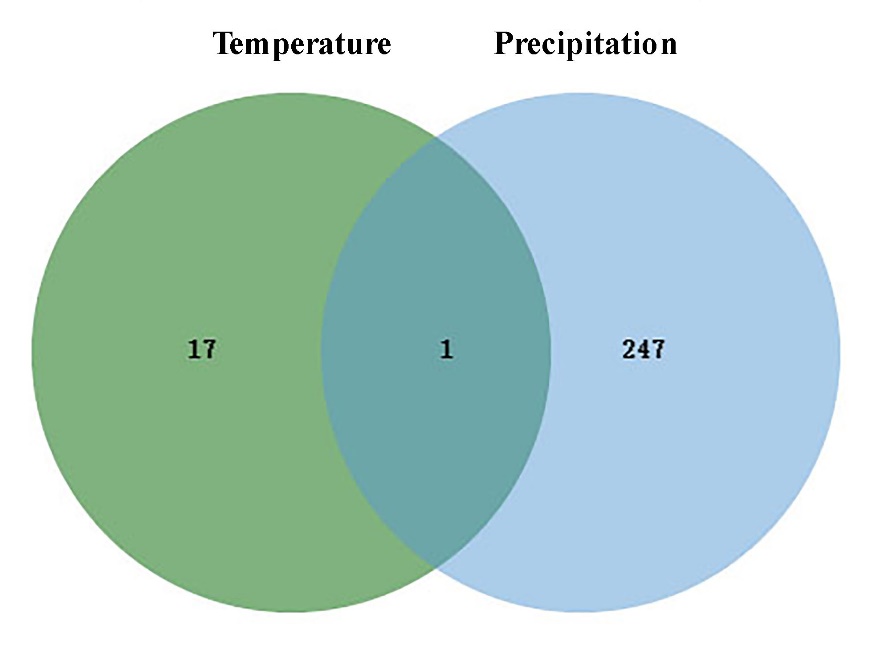


Figure S12. Venn of temperature and precipitation-related core adaptive variables in *P. pruinosa* populations.
